# Society Meetings

**Published:** 1901-09

**Authors:** 


### SOCIETY MEETINGS.

The Medical Society of the County of Cattaraugus and the Thomas J. King Medical Club held a joint meeting at Hazelmere Inn, Lime Lake, N. Y., Tuesday, August 6, 1901, under the presidency of Dr. James A. Taggart, of Salamanca, N.Y. Dr. William C. Krauss, of Buffalo, read a paper entitled, Toxic dosage in the treatment of some nervous diseases; Dr. Wm. B. Johnston, of Ellicottville, one entitled, Acute poliomyelitis, and Dr. Wm.A. MacFarlane, of Springville, also contributed a paper.

The Mississippi Valley Medical Association will hold its next annual meeting under the presidency of Dr. A. H. Cordier, of Kansas City, at the Hotel Victory, Put-in-Bay, Ohio, Thursday, Friday and Saturday, September 12, 13, and 14, 1901. The railroad rates for this meeting are a one-fare rate by way of Cleveland, which will enable those taking advantage of these rates to obtain an extension of tickets to October 8th for attendance upon the Buffalo exposition. A one-and-a-third fare rate on the certificate plan will be in effect via Detroit, Sandusky and

Toledo, with extension of return limit for only three days after the meeting. The address in Medicine will be made by Dr. Frank Billings, of Chicago; the address in Surgery by Dr. Reginald Sayre, of New York City. The annual banquet will be held on the evening of the first day, September 12th; the second evening will be assigned to the reading of several papers with stereopticon exhibits and demonstrations; the president's address and the annual orations will be delivered on the three mornings of the meeting. The profession is cordially invited to attend.

---

The Southern Surgical and Gynecological Association will hold its next annual meeting at Richmond, Va., Tuesday, Wednesday and Thursday, November 12, 13 and 14, 1901, under the presidency of Dr. Manning Simmons, of Charleston, S. C. The secretary, Dr. W. E. Haggard, Jr., of Nashville, Tenn., has issued a preliminary circular inviting contributions of papers. The city chosen for the coming meeting is both interesting and hospitable and a most successful group of sessions may be anticipated. Dr. George Ben Johnston, of Richmond, is chairman of the committee of arrangements, which means that everything possible will be done for the convenience and comfort of the visitors.

---

The Medical Association of Central New York is holding a well attended and interesting meeting under the presidency of Dr. Lucien Howe, of Buffalo, while these pages are going through the press.

---

The Association of American Medical Editors, at its annual meeting, at St. Paul, June 5, 1901, elected officers for the ensuing year as follows: President, Alex. J. Stone, of St. Paul; vice-president, Burnside Foster, of St. Paul; secretary and treasurer, O. F. Ball, of St. Louis. The executive committee appointed for the ensuing year consisted of Drs. Gould, Mathews, Lillie, Fassett, and Marcy. The next meeting will be held at Saratoga Springs, N.Y., in June, 1902.

---

The American Public Health Association representing the United States of America, the Dominion of Canada, the Republic of Mexico, will hold its 29th annual meeting at Buffalo, N. Y., Monday, Tuesday, Wednesday, Thursday and Friday, September 16, 17, 18, 19 and 20, 1901.

The chairmen of local committees are as follows: Arrangements, Henry Reed Hopkins; finance, D. W. Harrington; membership, Ernest Wende; reception and entertainment, Charles Cary; printing and publishing, William Warren Potter; bacteriology and exhibits, William G. Bissell; halls, Walter D. Greene; information, Dewitt H. Sherman.

The executive committee has selected the following topics for consideration, but papers will be received upon other sanitary subjects. General meeting—I. The pollution of public water supplies ; II. The disposal of refuse material ; III. Animal diseases and animal food; IV. Car sanitation; V. Etiology of yellow fever ; VI. Steamship and steamboat sanitation ; VII. Relation of forestry to the public health; VIII. Demography and statistics in their sanitary relation; IX. Cause, prevention and duration of infectious diseases; X. Public health legislation; XI. Cause and prevention of infant mortality; XII. Disinfectants and disinfection; XIII. National leper homes; XIV. Dangers to the public health from illuminating and fuel gas; XV. Transportation of diseased tissue by mail; XVI. The teaching of hygiene and granting of diploma of doctor of public health ; XVII. School hygiene ; XVIII. Sanitary aid societies.

Section on bacteriology and chemistry—I. On standard methods of water analysis; II. Bacteriology of milk in its sanitary relations; III. Variations of the colon bacillus in relation to public health; IV. Exhibition of laboratory apparatus and appliances for teaching hygiene.

A daily program will be issued each morning giving the titles of papers, reports, and the like, that will be presented, with such other information as may be of interest in connection with the work of the day.

The headquarters of the executive committee will be at the Niagara Hotel. A meeting of the committee will be held in one of the parlors on Monday, September 16, at 10 a.m. Subsequent meetings of the committee will be held daily at such hours as hereafter may be determined.

The advisory council will meet Thursday, September 19, the hour and place to be announced by the president, for the purpose of acting as a nominating committee of officers for the ensuing year, to select the place of the next annual meeting, and to consider such questions and make such recommendations as shall best secure the objects of the association.

On account of the Pan-American Exposition all railroads have granted rates to Buffalo of one fare, plus one dollar, for the round trip. It is possible that still lower rates will be offered before the time of the meeting.

The Niagara Hotel will be the headquarters of the association. This hotel is on Porter Avenue, between Seventh Street and Front Avenue. It contains 210 rooms. Rates are $2.00 a day and upwards—European plan—for one person.

The place of meeting will be at the Armory of the 74th Regiment, one block from the Niagara Hotel. The general association will open its meetings in the Armory on Tuesday morning, September 17, at 10 o'clock. An opening address will then be given by Dr. Stephen Smith, of New York, first president of the Association. The public meeting will occur Tuesday evening, September 17, at 8 o'clock. Addresses of welcome will be given, after which the annual address of the president of the association, Dr. Benjamin Lee, will be read. The section on bacteriology and chemistry will hold its meetings at the Armory, beginning Monday, September 16.

The meetings of the association are open to the public. Many valuable papers have been secured which, with the reports from special committees, insures an interesting program, and all persons, of whatever profession or occupation, are cordially invited to be present.

Further information concerning local matters, if desired, may be obtained by addressing Dr. H. R. Hopkins, chairman local committee of arrangements, Buffalo, N.Y.

The officers for 1900-1901, are: president, Dr. Benjamin Lee, Philadelphia; first vice-president, Rudolph Hering, C. E., New York; second vice-president, Dr. John N. Hurty, Indianapolis; secretary, Charles O. Probst, Columbus; treasurer, Dr. Henry D. Holton, Brattleboro.

NOTE—At the last annual meeting of the Conference of State and Provincial Boards of Health of North America, it was voted to hold their next meeting in connection with the meeting of the American Public Health Association. Accordingly the conference has arranged to meet at the Cataract House, Niagara Falls, on Friday and Saturday, September 13 and 14, 1901.

---

THE American Association of Obstetricians and Gynecologists will hold its fourteenth annual meeting at the Hotel Hollenden, Cleveland, O., Tuesday, Wednesday and Thursday, September

17, 18 and 19, 1901, under the presidency of Dr. William E. B. Davis, of Birmingham, Ala. The committee of arrangements is composed of Dr. M. Rosenwasser, 722 Woodland Avenue and Dr. William H. Humiston, 122 Euclid Avenue, Cleveland, either of whom may be addressed concerning rooms or other local information regarding the meeting.

The following list of papers is offered:

1. The president's address, William E. B. Davis, Birmingham.

2. Indications for the combined vagino-abdominal operation of hysterectomy, Rufus B. Hall, Cincinnati.

3. Title to be announced, Henry D. Ingraham, Buffalo.

4. A method for suspension of the uterus, Robert T. Morris, New York.

5. Tubercular peritonitis, experimental and clinical, John B. Murphy, Chicago.

6. Report of a case of acute pancreatitis and fat necrosis, Edward J. Ill, Newark.

7. Pelvic and abdominal tumors complicating pregnancy, with report of cases, Rufus B. Hall, Cincinnati.

8. Pathology and treatment of hourglass stomach, with report of two cases, Charles G. Cumston, Boston.

9. Early operations in appendicitis and method, Joseph Price, Philadelphia.

10. Vaginal hysterectomy, with four and a half months' pregnancy and closed cervix, J. Henry Carstens, Detroit.

11. The undeveloped uterus, C. L. Bonifield, Cincinnati.

12. An interesting case of tubo-abdominal pregnancy, Wm. H. Humiston, Cleveland.

13. Title to be announced, Walter B. Dorsett, St. Louis.

14. Some rare and odd cases and experiences in pelvic and abdominal surgery; the lessons they teach, C. C. Frederick, Buffalo.

15. Report of a case of ruptured tubal pregnancy, Webb J. Kelly, Piqua, O.

16. Diseases and injuries of the cervix uteri and their treatment, Joel W. Hyde, Brooklyn.

17. Gallstones in the common duct; remarks upon their frequency, diagnosis and treatment, L. H. Dunning, Indianapolis.

18. Title to be announced, James F. W. Ross, Toronto.

19. Extrauterine pregnancy; report of cases with specimens, George S. Peck, Youngstown.

20. Is Cesarean section justifiable in placenta previa? E. Gustav Zinke, Cincinnati.

21. Retrodisplacements in young girls; their frequency and best methods of treatment, H. E. Hayd, Buffalo.

22. Some reflections on ectopic gestation, David Tod Gilliam, Columbus.

23. Some forms of disease involving the uterine appendages, Augustus P. Clarke, Cambridge.

24. The mechanical, or combined plastic and mechanical treatment of retrodeviations of the womb, M. Rosenwasser, Cleveland.

25. New method of opening the abdomen in gynecological surgery, Charles G. Cumston, Boston.

26. Indications, technic, and remote results of salpingostomy, and of resection and ignipuncture of the ovaries, with tables of 104 cases, A. Goldspohn, Chicago.

27. Sarcoma of the breast, with report of a case, Edwin Ricketts, Cincinnati.

28. Some observations on the surgery of the spleen, Lewis S. McMurtry, Louisville.

29. The rôle of nephrotomy and nephrectomy in suppurative and malignant diseases of the kidney, Charles A. L. Reed, Cincinnati.

30. Pregnancy in bicornate uterus; rupture in third month; operation; recovery; specimen, Miles F. Porter, Fort Wayne.

31. Galvanism as a remedy for uterine hemorrhages, Edwin Walker, Evansville.

32. Papilloma of the vulva, with specimens, Edward J. Ill, Newark.

33. The need of constitutional treatment in gynecological cases, Walter B. Chase, New York.

---

The American Electro-therapeutic Association will hold its 11th annual meeting at Buffalo, September 24, 25 and 26, 1901.

The following-named are the officers and committees for 1901-1902: President, Ernest Wende, 471 Delaware Ave., Buffalo, N.Y.; first vice-president, Frederic H. Morse, Melrose, Mass.; second vice-president, D. R. Brower, Chicago, Ill.; secretary, George E. Bill, 255 North St., Harrisburg, Pa.; treasurer, R. J. Nunn, 119 York St., Savannah, Ga.

Executive council—Robert Newman, 148 W. 73d Street, New York City; G. Betton Massey, 1831 Chestnut St., Philadelphia,

Pa.; Charles R. Dickson, 296 Sherbourne St., Toronto, Canada; Frederic Schavoir, 8 Atlantic St., Stamford, Conn; F. B. Bishop, Washington, D.C.; W. H. White, Boston, Mass.

Committee on arrangements—Henry R. Hopkins, chairman, 433 Franklin St.; William Warren Potter, 284 Franklin St.; W. D. Greene, 385 Jersey St.; Newcomb Carlton, C. E., 109 White Building; Mr. Augustus Schneider, 574 West Ave.; A. W. Bayliss, 529 Spring St.; Edward Clark, 2268 Main St.; Willis G. Gregory, 530 Main St.; Franklin C. Gram, 46 Glenwood Ave.; Julius Ullman, 400 Fianklin St., all Buffalo, N. Y.

